# A Computational Model of Mechanical Stretching of Cultured Cells on a Flexible Membrane

**DOI:** 10.21203/rs.3.rs-8653612/v1

**Published:** 2026-02-14

**Authors:** Miles W. Massidda, David Ashirov, Andrei Demkov, Aidan Sices, Aaron B. Baker

**Affiliations:** University of Texas at Austin; University of Texas at Austin; University of Texas at Austin; University of Texas at Austin; University of Texas at Austin

**Keywords:** mechanobiology, computational cell modeling, chromatin deformation, mesenchymal stem cells, cellular mechanotransduction, substrate strain, mechanical stretch, multiscale modeling, tissue engineering

## Abstract

In this study, we developed a computational model of a cell being stretched on a flexible membrane, a configuration that matches many in vitro experimental systems studying the effects of mechanical stretch on cultured cells. Using this model, we explored the complex patterns of stresses and strains present in the cell during dynamic stretching. We linked these intracellular stresses to a simple model of chromatin deformation to provide a rough estimate of chromatin reconfiguration resulting from nuclear strain. Together, this multiscale model of cell stretching offers a first-order approximation of cellular strain responses to dynamic substrate deformation. Our simulations identified an optimal range of applied strain that induces chromatin distention without causing cellular damage. This computationally determined optimal strain range aligns with recent experimental findings from our laboratory, where the same strain levels were shown to maximize nuclear localization of Yap/Taz and reduce senescence in mesenchymal stem cells (MSCs). These results provide a computational framework for understanding cellular responses to mechanical stimuli, potentially optimizing experimental designs and advancing the understanding of mechanobiology in stem cell research and tissue engineering applications.

## Introduction

In recent years, mechanical forces have emerged as critical regulators of biological processes across multiple scales, from individual cells to whole organisms. These forces influence fundamental cellular behaviors such as proliferation, differentiation, and migration, as well as tissue formation and organ function.^1–4^ In tissue engineering and regenerative medicine, mechanical cues are increasingly recognized for their ability to direct tissue maturation and modulate the efficacy of regenerative therapies.^5–8^

Due to the challenges of controlling mechanical forces in vivo, in vitro models are essential for isolating and studying specific mechanical stimuli under well-defined conditions. A widely used experimental approach involves culturing cells on flexible membranes that can be subjected to controlled mechanical stretch.^9–12^ This setup has been instrumental in advancing our understanding of mechanobiology. However, many studies assume that cells experience the same strain as the substrate, an assumption that overlooks the complex intracellular mechanical environment. Measuring strain within cells remains technically challenging, and dynamic stretching introduces additional complexity due to the viscoelastic properties of cells, which can cause significant differences between substrate and cellular strains.^13–15^

At the heart of mechanobiological regulation is the deformation of the nucleus and chromatin, which plays a pivotal role in mechano-transduction pathways that influence cell phenotype, epigenetic regulation, and gene expression.^16–21^ Mechanical forces transmitted from the extracellular matrix and cytoskeleton to the nuclear envelope and chromatin can induce chromatin stretching, leading to changes in transcriptional activity, histone modifications, and chromatin organization.^16,20–22^ Our previous work demonstrated that specific dynamic mechanical stretches induce vascular differentiation in mesenchymal stem/stromal cells (MSCs) and mitigate cellular senescence while enhancing DNA damage repair.^23–25^ Remarkably, these mechanical stimuli impart a lasting cellular memory, with phenotypic effects persisting for weeks following mechanical conditioning and implantation. We proposed that mechanical stretch induces chromatin distention, exposing cryptic DNA damage sites that facilitate repair and rejuvenation.^23^

The objective of this study was to develop a computational model to quantify the stresses and strains experienced by cells cultured on flexible membranes subjected to dynamic mechanical stretch. Building on our prior findings emphasizing the importance of physiological dynamic waveforms,^10,23–25^ we compared the effects of standard sinusoidal stretch with a brachial artery waveform that mimics physiological cardiac cycle stretch. We evaluated a range of dynamic strains commonly used in experimental studies. Integrating recent insights into chromatin mechanics,^26^ we linked our mechanical model to a simplified chromatin deformation framework to provide a foundational understanding of how mechanical stretch regulates chromatin structure in cultured cells.

## Methods

### Computational model of cell stretching on a flexible membrane.

The computational model simulated the mechanical stretch of a cell attached to a flexible membrane. The mechanical and geometric parameters of the cell were taken from prior studies based on typical values for MSCs (Fig. 1A).^27–29^ The cell geometry was taken to be a half ellipsoid with an elliptical attachment to the surface. The nucleus geometry is represented as a smaller ellipsoid embedded within the cell (**SI** Fig. 1A, B). The model was defined by three main mechanical components: (1) The cell membrane as a viscoelastic layer with an elasticity of 2.98 kPa and viscosity of 4.01 kPa·s; (2) The cytoplasm with elasticity of 4 kPa and viscosity of 5.43 kPa·s; and (3) The nucleus with elasticity of 2.01 kPa and viscosity of 0.97 kPa·s (Fig. 1A; taken from literature values).^30^ The simulation of cell deformation was performed using COMSOL Multiphysics (Comsol, Inc., Burlington, MA; Version 6.0). The mechanics of the cytoplasm and nucleus were treated as a viscoelastic material with a Kelvin-Voigt model.^30^ The cell membrane was modeled using a viscoelastic membrane model. Cell model geometry was meshed using the parameters described in Fig. 1A. The geometry was meshed with a maximum element size of 0.99 μm and a maximum element growth rate of 1.4. The mesh near the edges of the cell compartments were refined to achieve a higher resolution, with a maximum element size of 0.159 μm **(Supplementary Fig. 1C-F)**. To apply strain to the cell, biaxial strain was applied to the bottom surface of the cell, assuming perfect coupling with the flexible membrane strain. The strain waveform function used was either a simple sine wave or a brachial wave form that simulates the complex distension of the brachial artery during the cardiac cycle (as described in our previous work).^31^

The transient Generalized Alpha Method was used for time-stepping with an upper limit on the time step size equal to 1/50 of the period T. The iterative Newton solver with a constant damping factor was applied. Simulation iterations were terminated when the estimated solution-based relative error is less than the specified relative tolerance of 0.001. PARDISO (Parallel Direct Sparse Solver) was used as the direct linear solver used for solving sparse linear systems of equations. The Function Sweep and Parametric Sweep were embedded into the main COMSOL modeling file. Function Sweep was used as the top-level for-loop, computing the solutions for sine and brachial waveforms. Parametric sweep was used as the nested for-loop, computing the solutions for all combinations of mechanical loading frequency and maximal strain amplitude.

### Model of chromatin deformation.

A recent study created a computational model of an axially stressed chromosome using a course-grained data-driven energy function (Minimal Chromatin Model; MiChroM).^32^ Using this simulation, they found that the mechanical response of the chromosome to sudden stress was well fit by a viscoelastic Kelvin-Voight: ϵ

$$\epsilon^{\prime }\left(t\right)=-1*\left[\frac{E}{\eta }\epsilon_{0}\right]+\frac{\sigma \left(t\right)}{\eta }$$

where *ε* is strain, *E* is the elastic modulus of the chromosome, *η* is the viscous damping constant, and *σ* is the input stress.^33^ Following the MiChroM model of chromatin under stress, we used *E* = 3.82 Pa and *η* = 68.81 N·s/m for interphase chromosomes, and *E* = 611.63 Pa and *η* = 733.95 N•s/m for mitotic chromosomes.^32^ To give a rough estimate of the forces applied to the chromatin, we calculated the spatial average stress in the nucleus and calculated the effects of this dynamic stress on the simplified model of chromatin using the viscoelastic model. The model assumes that all input stress is delivered is directly to a chromosome in interphase or mitosis. This likely overestimates the amount of stress on the chromatin and provides the upper limit of the forces applied to the chromatin.

## Results

### Computational model of a cell being stretched on flexible membrane.

**Figure 2:**
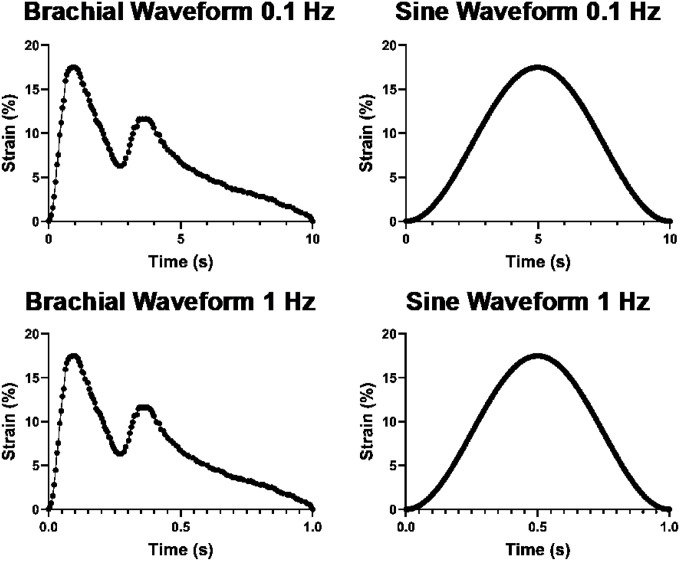
The strain profile of mechanical waveforms simulated in the computational model. The brachial waveform is a waveform that mimics the stretch of the brachial artery during the cardiac cycle (brachial loading) shown at 0.1 Hz or 1 Hz frequency of loading and can be scaled to different maximal strain and frequency of loading. The sinusoidal waveform is a commonly used strain waveform in experimental studies.

We created a computational model of a cell being stretching on a flexible membrane using a generalized geometry for the cell and nucleus taken from prior studies.^27–29^ The mechanical properties for the generalized cell was taken from a prior experimental study.^30^ The model included continuum models of the cytoplasm and nucleus that used viscoelastic properties consistent with prior experimental studies.^30^ In addition, a viscoelastic shell model was used for the cell membrane.^30^ Strain was applied to the cell through forced expansion of the basolateral surface of the cell, assuming the strain of the basolateral surface of the cells matched that of the flexible membrane. Dynamic strain waveforms of stretch were applied to cell, ranging from 2.5 to 17.5% maximal strain in increments of 2.5%. In addition, these strains were applied in a physiological waveform modeling the stretch of the brachial artery scaled to different amounts of maximal strain (“brachial waveform”; Fig. 2A, B) or sine wave of strain commonly used in many studies (Fig. 2C, D). In addition, we applied these combinations of strain at 0.1 Hz or 1 Hz frequency of loading.

### Nuclear strain is differentially dependent on the waveform, magnitude and frequency of applied stretch.

**Figure 3:**
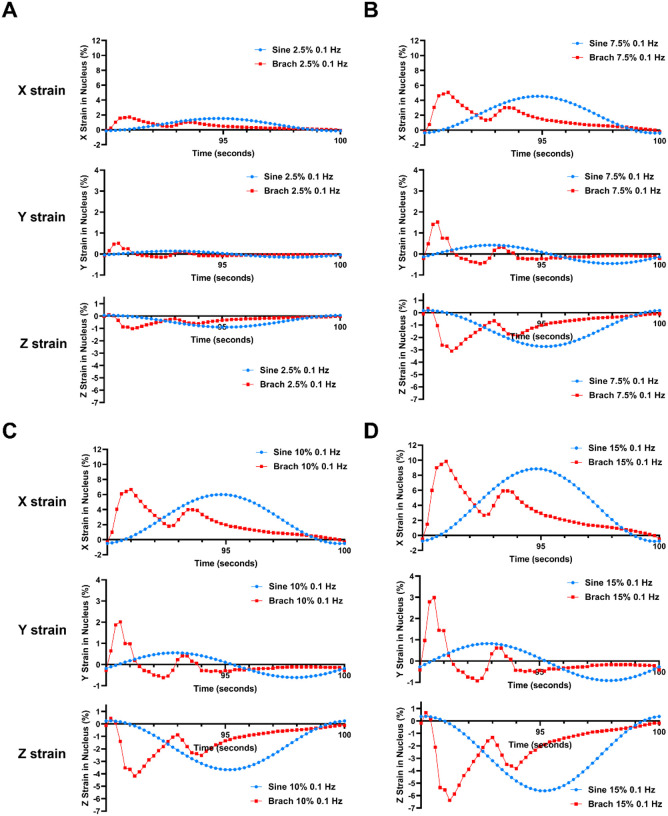
The average nuclear strain profile across the final cycle of loading demonstrates multi-dimensional generation of tensile and compressive strain. The average nuclear strain in the X, Y, and Z dimensions across cycle 10 were analyzed for 0.1 Hz sine and brachial waveforms of **(A)**2.5% maximal strain amplitude, **(B)** 7.5% maximal strain amplitude, **(C)**10% maximal strain amplitude, and **(D)** 15% maximal strain amplitude.

To examine how applied strain to the basolateral surface of the cell led to strain within the nucleus, we examine the average strain in the x, y and z directions of the nucleus under the various conditions. In our model, the x direction is long axis of the cell while the y direction is the short axis in the plane of the applied strain. In the x direction, we observed primarily tensile strains that mimicked the applied strain through the membrane (Fig. 3; **Animated Supplemental Fig. S2**). In the y direction, we found a similar shape of the strain over time but with tensile and compressive strains. In the z direction, we found that there were primarily compressive strains with a similar waveform of strain over time.

### The proportion of strain in the nucleus scales linearly with the applied strain and is dependent on the frequency of loading and strain waveform.

**Figure 4:**
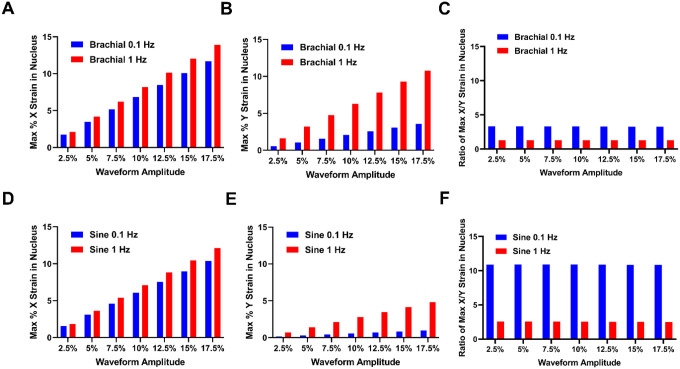
Maximal average nuclear strain under the various loading conditions. **(A, B)** Maximal nuclear strain for brachial waveforms in the **(A)** X dimension and **(B)** Y dimension. **(C)** The ratio of the maximal X/Y nuclear strain for brachial waveforms. **(D, E)** Maximal nuclear strain for sinusoidal waveforms in the **(D)** X dimension and **(E)** Y dimension. **(F)** The ratio of the maximal X/Y nuclear strain for sinusoidal waveforms.

The peak strains that reach the nucleus due to the loading of the dorsolateral surface of the cells scaled linearly with the applied strain (Fig. 4). With increased frequency of loading, more of the strain that was applied was transferred to the nucleus. For the x strain (long axis), the amount applied to the nucleus was somewhat higher for the high frequency of loading but in the y direction (short axis) the high frequence led to significantly higher strains (Fig. 4A, B, D, E). This has implications in how the nucleus is being deformed by the different types of strains. To examine this issue, we took the ration between the x and y directions (long/short axis ratio; Fig. 4C, F). A one-on-one ratio of x and y strains would imply a biaxial type loading of the ratio, while a higher ratio of x to y strain would imply stronger strains in the long axis (more uniaxial-like strain). The higher frequency (1 Hz) of loading had a more biaxial type strain application to the nucleus for both waveforms (Fig. 4C, F). In contrast, the lower frequency of loading (0.1 Hz) had a more uniaxial-like strain application. In addition, the ratio was dependent on the strain waveform, with the long to short axis strain ratio of the sine wave of approximately 11 and the brachial waveform ratio being around 3.5 (both a 0.1 Hz of loading). The ratio between the strains was independent of the magnitude of the applied for both frequencies of loading and waveforms.

### The nuclear strain profile caused by the first cycle of mechanical loading is significantly different from the strain caused by the remaining cycles of loading.

**Figure 5:**
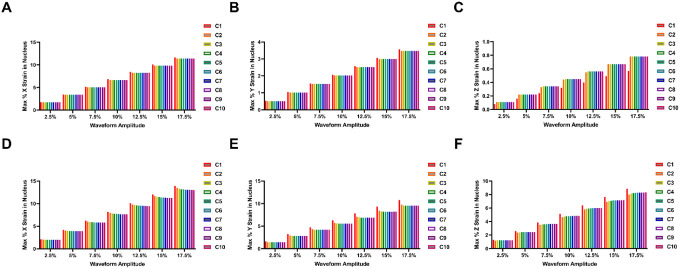
Effect of cyclic brachial loading on mechanical strain in the nucleus. **(A-C)** Simulated strain profile across 10 cycles of the brachial waveform at 0.1 Hz frequency of loading for nuclear strain in the **(A)** X, **(B)** Y, and **(C)** Z dimensions. **(D-F)** Simulated strain profile across 10 cycles of the brachial waveform at 1 Hz frequency of loading for nuclear strain in the **(D)** X, **(E)** Y, and **(F)** Z dimensions.

**Figure 6:**
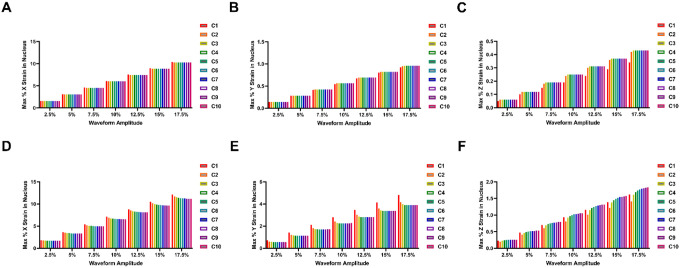
Effect of cyclic sinusoidal loading on mechanical strain in the nucleus. **(A-C)** Simulated strain profile across 10 cycles of the sinusoidal waveform at 0.1 Hz frequency of loading for nuclear strain in the **(A)** X, **(B)** Y, and **(C)** Z dimensions. **(D-F)** Simulated strain profile across 10 cycles of the sinusoidal waveform at 1 Hz frequency of loading for nuclear strain in the **(D)** X, **(E)** Y, and **(F)** Z dimensions.

In some experiments, researchers apply a single stretch event to the cells to understand the rapid mechanosensing pathways. To understand how the initial stages of stretching differ from continuous application of cyclic loading, we examined the strain in the nucleus as a function of cycle of loading over time (Fig. 5–6). For the first cycle, was a significant difference in the nuclear stain from the subsequent cycles and there was an exponential-type approach to the oscillatory steady state condition for most of the conditions and directions of strain. For all mechanical waveforms simulated, the nuclear strain in the X and Y dimensions is initially greater before falling in subsequent cycles. In the z direction (vertical) for 0.1 Hz frequency of loading, the initial strains were initially lower at cycle 1 and increased in subsequent cycles. For the z direction with 1 Hz of loading, a different pattern was exhibited over the cycles which was dependent on the waveform of loading. For these conditions, the brachial waveform had a higher initial nuclear strain follow by a drop in strain and an upward exponential approach to oscillatory steady state. For the sine waveform, there was a drop in nuclear strain after the first cycle but then there was an upward exponential decay towards a strain that was higher than the initial cycle.

### Estimation of chromatin stretch from nuclear deformation due to cycle mechanical loading.

**Figure 7:**
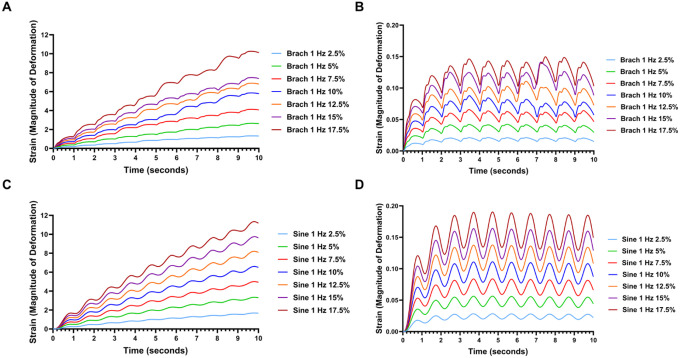
Estimated strain magnitude applied to chromatin in viscoelastic model at 1 Hz frequency of loading. **Es** Strain magnitude applied in the X dimension across 10 cycles of mechanical loading at 1 Hz for brachial waveform loading of **(A)** interphase chromatin and **(B)** mitosis chromatin. Strain magnitude applied in the X dimension across 10 cycles of mechanical loading at 1 Hz for sine waveform loading of **(C)** interphase chromatin and **(D)** mitosis chromatin.

**Figure 8:**
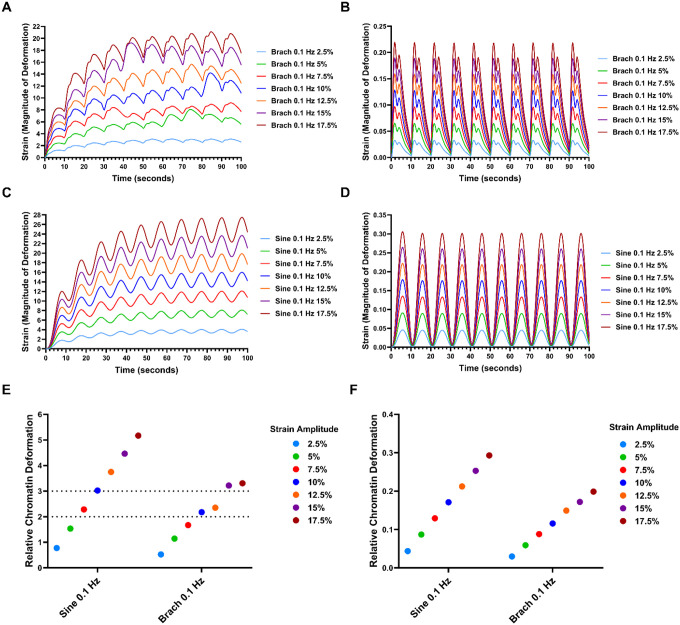
Estimated strain magnitude applied to chromatin in viscoelastic model at 0.1 Hz frequency of loading. Strain magnitude applied in the X dimension across 10 cycles of mechanical loading at 0.1 Hz for **(A)** brachial waveform loading of interphase and **(B)** mitosis chromatin. Strain magnitude applied in the X dimension across 10 cycles of mechanical loading at 0.1 Hz for **(C)** sine waveform loading of interphase and **(D)** mitosis chromatin. **(E)** Relative deformation of interphase chromatin across cycle 10 of simulated sine and brachial waveforms at 0.1 Hz frequency of loading. **(F)** Relative deformation of mitosis chromatin across cycle 10 of simulated sine and brachial waveforms at 0.1 Hz frequency of loading.

To link the consequences of nuclear deformation to the stretching of chromatin in the cells, we linked the continuum model to the resulting fit from a molecular model of chromatin stretching from a previous work.^32^ As the mechanics of chromatin is highly dependent on whether the cell is in interphase versus mitosis, we simulated the strain in the chromatin for both conditions. The interphase chromatin is more compliant than chromatin during the mitotic phase.^32^ For 1 Hz frequency of loading, the compliant nature of the interphase chromatin led to increasing strains over the ten cycles of loading (Fig. 7A, C). The mitotic chromatin, there was a more rapid approach to oscillatory steady state (Fig. 7B, D). At 0.1 Hz frequency of loading, there was a more rapid approach to oscillatory steady state in terms of the cycles of loading (Fig. 8A, C). The mitotic chromatin, in contrast had no lead in to the oscillatory steady state (Fig. 8B, D).

From a prior molecular simulation of chromatin stretching, the chromosome mechanically unravel when extensions reach roughly double its native length while damage to chromatin occurs at approximately three times its native length.^32^ To understand how the different loading conditions affected the chromatin, we estimated the relative chromatin deformation during the final cycle of loading for each the loading conditions (Fig. 8E, F). During interphase chromatin in cells loaded at 0.1 Hz, had chromatin extensions in the 2–3 fold extension range for 7.5% strain for the sine waveform and 10–12.5% strains for the brachial waveform of loading. For the mitotic stage, none of the strains induced the range of extension for unfolding chromatin. Our prior experimental work found that many of the peak effects of mechanical stretch on MSCs occurred in similar ranges of 7.5%−12.5%^25,34^ as well as in other cell types.^8,10^ These ranges of strains to the chromatin may provide sufficient stretch to alter gene transcription and chromatin structure, while not causing to mechanical damage.

## Discussion

In this study, we developed a multiscale computational model to investigate the mechanical behavior of a cell subjected to dynamic stretching on a flexible membrane, a widely used in vitro experimental configuration. Our model captures the complex intracellular stress and strain distributions during dynamic loading and links these mechanical cues to chromatin deformation within the nucleus. This approach provides a first-order approximation of how substrate deformation translates into nuclear and chromatin strain, offering insights into the mechanobiological processes that regulate cellular function.

Our computational model distinguishes itself from existing models by integrating multiscale mechanical behavior, from substrate deformation through cytoplasmic and nuclear viscoelasticity to chromatin-level strain estimation, within a single framework driven by physiologically relevant dynamic stretching waveforms. While earlier models have examined either cellular mechanics or nuclear deformation in isolation,^26,35–37^ or have coupled these components through simplified cytoskeletal representations,^38–40^ few models explicitly incorporate substrate-level mechanics and propagate strain estimates down to the chromatin scale under dynamic physiological loading conditions such as brachial artery stretch waveforms. By employing viscoelastic representations (Kelvin-Voigt) at both cytoplasmic and nuclear levels and propagating strain estimates to the chromatin scale, our model captures time-dependent mechanical responses critical for predicting cellular behavior under dynamic conditioning protocols.

Our findings demonstrate that nuclear strain is highly dependent on the waveform, magnitude, and frequency of applied stretch. Specifically, we observed that higher loading frequencies increase the proportion of strain transmitted to the nucleus, with distinct biaxial or uniaxial-like strain profiles emerging depending on the waveform and frequency. These results align with prior experimental observations that cellular responses vary with mechanical loading patterns and highlight the importance of considering physiological waveforms, such as the brachial artery stretch, in mechanobiological studies.^8,10,12,23–25^ The differential strain patterns in the nucleus likely influence downstream mechanotransduction pathways, including chromatin remodeling and gene expression regulation.

By integrating a simplified viscoelastic model of chromatin mechanics, our simulations estimate chromatin deformation under various loading conditions. The predicted chromatin strains fall within ranges previously associated with functional chromatin unfolding and gene transcription modulation, without reaching levels that cause mechanical damage.^16,22,41,42^ This supports the hypothesis that physiological mechanical stretch can induce beneficial chromatin remodeling, consistent with our prior experimental work showing enhanced vascular differentiation and reduced senescence in mesenchymal stem cells exposed to similar strain magnitudes.^23,25^ These findings underscore the potential of mechanical conditioning to modulate stem cell phenotype through nuclear and chromatin mechanotransduction.

Our study indicates that a minimum strain amplitude of 7.5% is necessary to mechanically unravel chromosomes under both sine and brachial loading conditions. Previous research has shown that chromosomes unravel when extended to approximately twice their native length.^32^ This unraveling is reversible, as the relaxation of applied force allows dissociated nucleosomes to reassemble, restoring chromatin organization.^43,44^ Based on this, we propose that specific mechanical loading regimes may reset chromatin architecture by stretching chromatin fibers, displacing nucleosomes, and facilitating reorganization during relaxation phases. In our simulations, waveforms with strain amplitudes below 7.5% induced oscillatory chromosome extensions of less than 1.5 times their native length at the start of each loading cycle, likely insufficient to trigger significant nuclear reorganization or sustained changes in transcription. Conversely, higher strain amplitudes exceeding 15% caused chromosome extensions of 3 to 4 times their native length per cycle, which could potentially damage critical mechanosensitive structures such as the cytoskeleton and the LINC complex.^45–47^ Thus, mid-range strain amplitudes between 7.5% and 12.5% may provide an optimal balance, inducing sufficient nuclear deformation to mechanically unravel chromosomes without causing permanent damage or apoptosis. This balance may underlie the enhanced regenerative properties and nuclear Yap/Taz signaling observed in mesenchymal stem cells exposed to moderate strain levels, as reported in our previous work.^31^

While our model advances understanding of cellular mechanobiology, there are several limitations that warrant consideration. The simplified geometry and mechanical properties, although based on experimental data, do not capture the heterogeneity and complex substructures of real cells and the nucleus. The assumption of perfect coupling between the cell and substrate likely overestimates strain transmission, and the chromatin model provides only an upper-bound estimate of deformation. We also assumed constant mechanical properties, adhesion and coupling during the loading. In true physiological conditions, there is dynamic regulation of the mechanical properties, cell structure and dynamic changes in coupling/de-coupling interacting between the subcellular components and between the cell and the extracellular matrix.^48^ These effects would likely grow in their effects over time as the cell adapts to its mechanical conditions. Future work incorporating more detailed nuclear architecture, active cytoskeletal remodeling, and real-time chromatin dynamics will enhance model fidelity.

Overall, our computational framework integrates mechanical behavior from substrate deformation to chromatin-level strain under physiologically relevant dynamic loading. By quantifying strain propagation across scales, the model identifies mechanical regimes that promote functional chromatin remodeling while avoiding cellular damage. This framework can guide the optimization of mechanical conditioning protocols in stem cell research and tissue engineering.

## Supplementary Material

Supplementary Files

This is a list of supplementary files associated with this preprint. Click to download.

- MechanoModelingPaperSIStorybook05.pptx

## Data Availability

All data generated or analyzed during this study are included in this published article and its supplementary information files.
